# Factors associated with AD8-defined high cognitive risk in community-dwelling middle-aged and older adults: a cross-sectional study

**DOI:** 10.3389/fpubh.2026.1927564

**Published:** 2026-10-29

**Authors:** Yuxin Ou, Xixian Yang, Yunshan Wang, Hongpeng Wu

**Affiliations:** 1 Department of Clinical Laboratory, Shandong Provincial Hospital Affiliated to Shandong First Medical University, Jinan, Shandong, China; 2 Department of Radiology, Shandong Provincial Hospital Affiliated to Shandong First Medical University, Jinan, Shandong, China

**Keywords:** AD8 positivity, associated factors, cognitive impairment screening, cross-sectional study, middle-aged and older adults

## Abstract

**Background:**

With the accelerating global population aging, cognitive dysfunction has become an important public health challenge among middle-aged and older adults. The Ascertain Dementia 8-item Informant Questionnaire (AD8) is a simple and sensitive screening tool for identifying individuals at risk of cognitive impairment in community settings. However, evidence regarding factors associated with AD8-defined high cognitive risk (AD8 positivity) among community-dwelling middle-aged and older adults remains limited. This study aimed to investigate factors associated with AD8 positivity in a large community-based population.

**Methods:**

A total of 7,601 participants were included in the descriptive and univariate analyses. All 14 candidate variables were entered into the multivariable logistic regression model, which was fitted using complete cases (*n* = 7,293). Household economic status was retained *a priori* based on previous epidemiological evidence.

**Results:**

Among the 7,601 participants, 6,116 (80.5%) were classified as having AD8 positivity. Univariate analyses showed that thirteen of the fourteen candidate variables were significantly associated with AD8 positivity. Multivariable logistic regression identified nine factors independently associated with AD8 positivity. Advanced age, female sex, marital status, poor self-rated health, depressive symptoms, and sleep disturbances were positively associated with AD8 positivity, whereas higher educational attainment and regular physical activity were negatively associated with AD8 positivity (indicating a lower risk). In addition, household economic status remained significantly associated with AD8 positivity after adjustment for potential confounders.

**Conclusion:**

AD8 positivity was highly prevalent among community-dwelling middle-aged and older adults in this study population. Age, sex, marital status, educational level, household economic status, regular physical activity, self-rated health, depressive symptoms, and sleep disturbances were independently associated with AD8 positivity. These findings may help identify vulnerable populations and provide evidence for community-based cognitive health management and early intervention strategies.

## 1 Introduction

The occurrence of cognitive dysfunction is influenced by multiple sociodemographic, lifestyle and health-related factors (1, 2). Cognitive decline and dementia have been linked to advanced age, female sex, low educational attainment, depressive symptoms, sleep problems, chronic disease burden, and physical inactivity, according to prior research (3–6). Understanding these potentially modifiable factors is important for identifying vulnerable populations and developing targeted prevention strategies (7, 8).

Although numerous studies have investigated factors related to cognitive impairment, several gaps remain. Primarily, many existing studies have been conducted among clinical patients, hospitalized populations, or individuals with confirmed cognitive disorders, which may limit the generalizability of their findings to community-dwelling populations (9, 10). Furthermore, previous research has often focused on individual risk factors, whereas the combined influence of demographic characteristics, lifestyle behaviors, physical health and psychological factors has been less frequently examined within the same analytical framework (11, 12). Lastly, evidence regarding factors associated with AD8-defined high cognitive risk (AD8 positivity) in community settings remains relatively limited, particularly among middle-aged and older adults in China (13).

As a brief and practical screening instrument, the Ascertain Dementia 8-item Informant Questionnaire (AD8) is widely used for the early identification of individuals with potential cognitive abnormalities in community and primary healthcare settings (14–16). Exploring the factors associated with AD8 positivity may help identify vulnerable groups at an early stage and provide evidence for community-based cognitive health management and preventive interventions (17).

To address the limited evidence regarding associated factors for early cognitive risk, we utilized the AD8 as a practical and highly sensitive instrument for large-scale screening. Accordingly, using baseline data from the Taierzhuang cohort, this study aimed to investigate the multidimensional factors independently associated with AD8 positivity among middle-aged and older adults. The Taierzhuang cohort, representing a rural-to-urban transitional population with a high proportion of low educational attainment and an aging demographic structure, provides a highly relevant setting for early cognitive screening research. Through multivariable logistic regression analysis, we sought to provide actionable evidence to inform the development of targeted cognitive health promotion and preventive strategies.

## 2 Materials and methods

### 2.1 Study population

Data for this study were derived from the baseline survey of the Taierzhuang cohort. The screening process for participant inclusion and exclusion criteria is presented in Figure 1. A total of 8,259 permanent community residents aged 50 years and above were initially enrolled in the baseline survey. The 8-item Dementia Screening Questionnaire (AD8) was utilized to preliminarily assess the cognitive status of all enrolled participants. To ensure data quality and minimize entry errors, all completed paper questionnaires were double-entered and cross-checked independently using EpiData software (version 3.1; EpiData Association, Odense, Denmark). Logic checks and rigorous data cleaning procedures were systematically performed to resolve any discrepancies against the original records prior to database locking. A total of 8,259 permanent community residents aged 50 years and above were initially enrolled in the baseline survey. During the initial data screening, 658 participants were excluded due to missing core demographic data, behavioral indicators, or primary health-related predictors, leaving 7,601 participants for descriptive and univariate analyses. Among these 7,601 participants, a further 308 were excluded from the multivariable analyses specifically because of missing values in one or more additional covariates required for the adjusted models. Consequently, the multivariable logistic regression and sequentially adjusted analyses were conducted using 7,293 complete cases. The reasons for exclusion at each stage are detailed in the participant flow diagram (Figure 1). According to the scoring criteria of the AD8 scale, an AD8 score ≥2 was defined as AD8 positivity. The initial analytical population (*N* = 7,601) was stratified into the low-risk group (AD8 < 2, *n* = 1,485) and the AD8 positivity group (AD8 ≥ 2, *n* = 6,116). Complete case analysis was adopted to handle missing values in the present study. Given that the missing rate of each candidate variable was less than 5% and the total proportion of excluded individuals across both stages was relatively small, multiple imputation was not adopted (18). Furthermore, as the proportion of excluded participants was relatively low, selection bias arising from participant exclusion is likely to be minimal.

**Figure 1:**
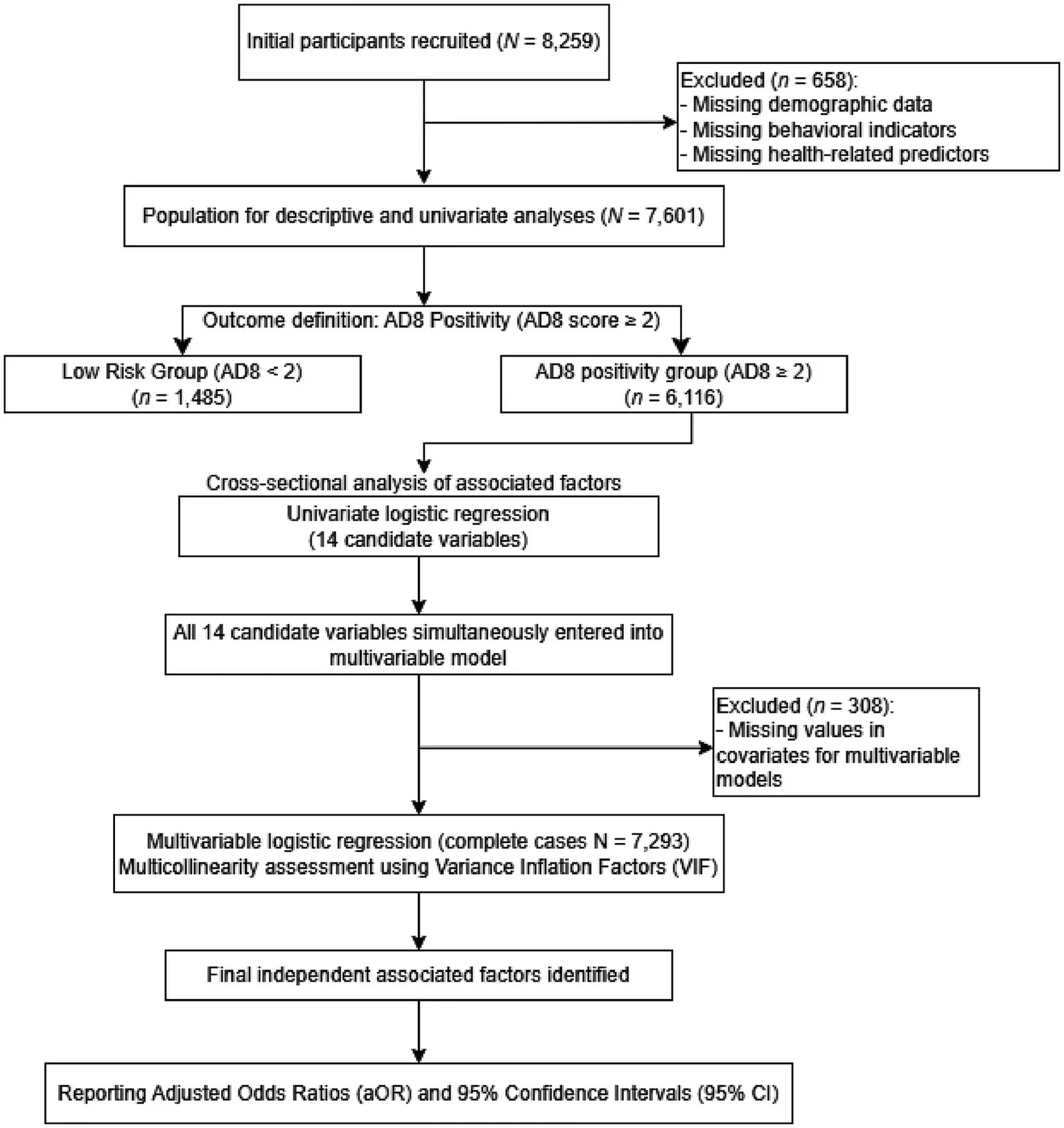
Flowchart of participant screening and study design. Legend: Participants from the Taierzhuang cohort aged ≥50 years were screened for eligibility. Individuals with missing demographic, behavioral, or health-related data were excluded. The final analytical population was stratified into two groups based on the AD8 assessment, with a score ≥2 defined as AD8 positivity. AD8, Ascertain Dementia 8-item Informant Questionnaire.

### 2.2 Outcome definition

The primary outcome of this study was AD8 positivity. The 8-item Informant Questionnaire (AD8) was used to assess participants’ cognitive status through structured, face-to-face interviews. Although originally designed as an informant-based tool, all participants in this study completed the questionnaire via self-report. The scale consists of 8 items evaluating self-perceived changes in memory, orientation, judgment, and daily functioning compared with their previous baseline level over the past several years. Each item receives a score of 0 if there is no decline or change, and 1 point if there is a decline in comparison to the prior condition, yielding a total score between 0 and 8. Referring to previous studies and clinically recognized criteria, AD8 positivity was defined as an AD8 total score of ≥2. This cut-off value has good sensitivity and specificity in community populations, and previous validation studies have confirmed that the self-reported AD8 reliably identifies early cognitive risk among middle-aged and older adults in China (16, 19).

### 2.3 Candidate variables

A total of 14 predefined variables covering demographic characteristics, lifestyle factors, and health-related indicators were included to develop the risk prediction model for AD8 positivity. The candidate variables were age, sex, educational attainment, household economic status, smoking and alcohol consumption, regular physical activity, body mass index (BMI), number of chronic diseases, self-rated health, depressive symptoms (measured by the CES-D, with a cut-off score ≥16) (20), anxiety symptoms (measured by the SAS, with a cut-off score ≥50) (21), sleep disturbances (measured by the PSQI, with a cut-off score >7) (22), and marital status. All standardized cut-off values were defined according to established clinical validation criteria in Chinese populations (23). In order to ensure the correctness of the data input for the model, all candidate predictors were gathered through in-person interviews and physical examinations during the baseline survey.

### 2.4 Univariate analysis

Univariate logistic regression analysis was conducted among the 7,601 valid participants to evaluate the associations between each of the 14 candidate variables and AD8 positivity (defined as AD8 score ≥2, coded as 1 for AD8 positivity and 0 for low risk). Crude odds ratios (ORs) and corresponding 95% confidence intervals (CIs) were calculated to examine the unadjusted associations.

### 2.5 Multivariable logistic regression

To ensure analytical rigor and minimize selection bias, all thirteen variables that showed significant associations in the univariate analysis (*p* < 0.05) were simultaneously entered into the multivariable logistic regression model. Additionally, household economic status was retained in the final model regardless of its univariate significance, given the strong prior epidemiological evidence supporting socioeconomic status as a fundamental determinant of cognitive health (24). For multicategorical variables, appropriate reference categories were specified before model fitting. The results of the multivariable analysis were reported as adjusted odds ratios (aORs) and corresponding 95% confidence intervals (CIs). At a significance level of two-tailed *p* < 0.05, factors independently associated with AD8 positivity were identified. Variance inflation factor (VIF) diagnostics were performed to assess potential multicollinearity among the independent variables. VIF values were examined for all predictors, and any elevated values were further investigated to assess their potential impact on model stability. Furthermore, the Hosmer–Lemeshow goodness-of-fit test was conducted to evaluate model calibration, which indicated a satisfactory model fit (*p* > 0.05). Exploratory interaction analyses were also performed among key demographic and health indicators, and no statistically significant interaction effects were observed.

### 2.6 Statistical analysis

Statistical analyses were performed using IBM SPSS Statistics version 30.0 (IBM Corp., Armonk, NY, United States) following data extraction from the locked database. Data visualization, including the construction of forest plots, was conducted using R software (version 4.5.3; R Foundation for Statistical Computing, Vienna, Austria) (25) with the forestplot package (26).

Before analysis, continuous variables were examined for normality. As most continuous variables did not follow a normal distribution, they were described as median and interquartile range (IQR), and comparisons between groups were performed using the Mann–Whitney *U* test. Categorical variables were presented as frequencies and percentages, and comparisons between groups were conducted using the chi-square test. All statistical tests were two-sided, and a *p*-value <0.05 was considered statistically significant. The reporting of this study followed the Strengthening the Reporting of Observational Studies in Epidemiology (STROBE) statement for cross-sectional studies (27).

## 3 Results

### 3.1 Participant characteristics

A total of 8,259 participants were initially recruited in the present study. After initial exclusions, 7,601 subjects were enrolled for descriptive and univariate analysis (Tables 1, 2), while 7,293 subjects with complete data were included in the multivariable models (Table 3). Baseline characteristics of these 7,601 subjects are summarized in Table 1. A notable finding of this study was the relatively high detection rate of AD8 positivity at 80.5% (6,116/7,601) in the study population. Because the AD8 is a highly sensitive screening instrument rather than a clinical diagnostic tool, this finding is discussed in detail in Section 4.6. Group comparisons based on AD8 screening results revealed that individuals with AD8 positivity exhibited a distinct demographic and health profile: they tended to be older, have lower educational attainment, and present poorer overall physical and mental health status across most evaluated indicators (all *p* < 0.05). Notably, household economic status was the only characteristic that showed no statistically significant difference between the two groups at the conventional threshold (*p* = 0.084).

**Table 1:** Baseline characteristics of the core modeling cohort stratified by cognitive status (*N* = 7,601).

Variables	Total (*N* = 7,601)	Low risk (*N* = 1,485)	High risk (*N* = 6,116)	Statistic	*p-*value
Age [years, Median (IQR)]	68.0 (60.0, 74.0)	63.0 (57.0, 70.0)	70.0 (62.0, 75.0)	*Z* = −19.930	<0.001
Sex, *n* (%)				*χ*^2^ = 77.615	<0.001
Male	3,242 (42.7)	784 (52.8)	2,458 (40.2)		
Female	4,359 (57.3)	701 (47.2)	3,658 (59.8)		
Educational level, *n* (%)				*χ*^2^ = 368.495	<0.001
Non-literate	3,548 (46.7)	407 (27.4)	3,141 (51.4)		
Primary school	1,777 (23.4)	356 (24.0)	1,421 (23.2)		
Junior high school	1,574 (20.7)	478 (32.2)	1,096 (17.9)		
Senior high school/Technical secondary school	538 (7.1)	190 (12.8)	348 (5.7)		
College and above	164 (2.2)	54 (3.6)	110 (1.8)		
Marital status, *n* (%)				*χ*^2^ = 119.183	<0.001
Unmarried	123 (1.6)	14 (0.9)	109 (1.8)		
Married	6,269 (82.9)	1,367 (92.4)	4,902 (80.5)		
Divorced/Widowed/Others	1,174 (15.5)	98 (6.6)	1,076 (17.7)		
Household economic status, *n* (%)				*χ*^2^ = 4.945	0.084
Good/Very good	1,640 (21.6)	349 (23.5)	1,291 (21.1)		
Average	5,402 (71.1)	1,021 (68.8)	4,381 (71.6)		
Poor/Very poor	559 (7.4)	115 (7.7)	444 (7.3)		
BMI [kg/m^2^, Median (IQR)]	23.9 (21.9, 26.0)	24.5 (22.5, 26.3)	23.7 (21.6, 26.0)	*Z* = −8.124	<0.001
Smoking status, *n* (%)				*χ*^2^ = 8.813	0.012
Never smoker	6,016 (82.2)	1,152 (79.5)	4,864 (82.8)		
Current smoker	1,176 (16.1)	269 (18.6)	907 (15.4)		
Former smoker	131 (1.8)	28 (1.9)	103 (1.8)		
Drinking status, *n* (%)				*χ*^2^ = 46.955	<0.001
Never drinker	6,037 (79.6)	1,087 (73.2)	4,950 (81.1)		
Current drinker	1,425 (18.8)	362 (24.4)	1,063 (17.4)		
Former drinker	125 (1.6)	36 (2.4)	89 (1.5)		
Regular exercise, *n* (%)				*χ*^2^ = 174.035	<0.001
No	4,601 (60.5)	676 (45.5)	3,925 (64.2)		
Yes	3,000 (39.5)	809 (54.5)	2,191 (35.8)		
Number of chronic diseases, *n* (%)				*χ*^2^ = 16.616	<0.001
0	4,387 (59.2)	907 (62.3)	3,480 (58.4)		
1	2,299 (31.0)	446 (30.6)	1,853 (31.1)		
≥2	725 (9.8)	103 (7.1)	622 (10.4)		
Poor self-rated health, *n* (%)				*χ*^2^ = 35.785	<0.001
No	7,138 (93.9)	1,444 (97.2)	5,694 (93.1)		
Yes	463 (6.1)	41 (2.8)	422 (6.9)		
Depressive symptoms (CES-D ≥16), *n* (%)				*χ*^2^ = 78.788	<0.001
No	6,795 (89.4)	1,422 (95.8)	5,373 (87.9)		
Yes	806 (10.6)	63 (4.2)	743 (12.1)		
Anxiety symptoms (SAS ≥50), *n* (%)				*χ*^2^ = 25.602	<0.001
No	7,071 (93.0)	1,426 (96.0)	5,645 (92.3)		
Yes	530 (7.0)	59 (4.0)	471 (7.7)		
Sleep disturbance (PSQI >7), *n* (%)				*χ*^2^ = 85.414	<0.001
No	5,412 (71.2)	1,202 (80.9)	4,210 (68.8)		
Yes	2,189 (28.8)	283 (19.1)	1,906 (31.2)		

The Mann–Whitney *U* test (*Z*-statistic) is used to compare continuous variables, which are displayed as median (interquartile range, IQR). The Chi-square test (χ^2^) is used to compare categorical variables, which are displayed as frequencies (percentages). An AD8 score of ≥2 was considered indicative of AD8 positivity.

BMI, Body Mass Index; CES-D, Center for Epidemiologic Studies Depression Scale; PSQI, Pittsburgh Sleep Quality Index; SAS, Self-Rating Anxiety Scale.

**Table 2:** Univariate logistic regression analysis of variables associated with AD8 positivity (*N* = 7,601).

Variables	Crude OR (95% CI)	*p*-value
Age (years)	1.07 (1.06–1.07)	<0.001
Sex		
Male	1.00 (Ref)	
Female	1.66 (1.49–1.87)	<0.001
Educational level		<0.001
Non-literate	1.00 (Ref)	
Primary school	0.52 (0.44–0.60)	<0.001
Junior high school	0.30 (0.26–0.35)	<0.001
Senior high school/Technical secondary school	0.24 (0.19–0.29)	<0.001
College and above	0.26 (0.19–0.37)	<0.001
Marital status		<0.001
Unmarried	1.00 (Ref)	
Married	0.46 (0.26–0.81)	0.01
Divorced/Widowed/Others	1.41 (0.78–2.55)	0.256
Household economic status		0.146
Poor/Very poor	1.00 (Ref)	
Average	0.96 (0.76–1.21)	0.751
Good/Very good	1.11 (0.90–1.38)	0.404
BMI (kg/m^2^)	0.94 (0.93–0.96)	<0.001
Smoking status		0.012
Never smoker	1.00 (Ref)	
Current smoker	0.78 (0.68–0.91)	0.003
Former smoker	0.83 (0.55–1.25)	0.523
Drinking status		<0.001
Never drinker	1.00 (Ref)	
Current drinker	0.65 (0.56–0.74)	<0.001
Former drinker	0.54 (0.37–0.80)	0.004
Regular exercise		
No	1.00 (Ref)	
Yes	0.47 (0.42–0.52)	<0.001
Number of chronic diseases		<0.001
0	1.00 (Ref)	
1	1.08 (0.95–1.23)	0.242
≥2	1.57 (1.26–1.96)	<0.001
Poor self-rated health		
No	1.00 (Ref)	
Yes	2.61 (1.88–3.62)	<0.001
Depressive symptoms (CES-D ≥16)		
No	1.00 (Ref)	
Yes	3.12 (2.40–4.06)	<0.001
Anxiety symptoms (SAS ≥50)		
No	1.00 (Ref)	
Yes	2.02 (1.53–2.66)	<0.001
Sleep disturbance (PSQI >7)		
No	1.00 (Ref)	
Yes	1.92 (1.67–2.21)	<0.001

This table presents the crude associations between candidate variables and AD8 positivity based on univariate logistic regression analyses. In the logistic regression analyses, AD8 positivity was coded as 1, and low risk was coded as 0. Odds ratios (ORs), 95% confidence intervals (CIs), and corresponding p values are reported.

OR, Odds Ratio; CI, Confidence Interval; Ref, Reference category.

**Table 3:** Multivariable logistic regression analysis of independent predictors for AD8 positivity (*n* = 7,293).

Variables	*β*	SE	Adjusted OR (95% CI)	*p*-value
Age (years)	0.048	0.004	1.049 (1.041–1.058)	<0.001
Sex				
Male	–	–	1.00 (Ref)	–
Female	0.383	0.08	1.467 (1.254–1.715)	<0.001
Educational level				<0.001
Non-literate	–	–	1.00 (Ref)	–
Primary school	−0.223	0.088	0.800 (0.674–0.951)	0.011
Junior high school	−0.542	0.09	0.582 (0.488–0.694)	<0.001
Senior high school/Technical secondary school	−0.804	0.119	0.447 (0.354–0.565)	<0.001
College and above	−0.427	0.197	0.652 (0.443–0.961)	0.03
Marital status				<0.001
Unmarried	–	–	1.00 (Ref)	–
Married	−0.805	0.311	0.447 (0.243–0.822)	0.01
Divorced/Widowed/Others	−0.436	0.329	0.647 (0.339–1.232)	0.185
Household economic status				<0.001
Poor/Very poor	–	–	1.00 (Ref)	–
Average	0.739	0.138	2.094 (1.599–2.743)	<0.001
Good/Very good	0.6	0.124	1.822 (1.430–2.322)	<0.001
BMI (kg/m^2^)	−0.011	0.01	0.989 (0.971–1.008)	0.249
Smoking status				0.529
Never smoker	–	–	1.00 (Ref)	–
Current smoker	0.104	0.094	1.110 (0.924–1.334)	0.266
Former smoker	0.081	0.251	1.084 (0.663–1.773)	0.747
Drinking status				0.457
Never drinker	–	–	1.00 (Ref)	–
Current drinker	0.022	0.089	1.022 (0.859–1.217)	0.804
Former drinker	−0.282	0.24	0.754 (0.471–1.208)	0.241
Regular exercise				
No	–	–	1.00 (Ref)	–
Yes	−0.534	0.064	0.586 (0.517–0.664)	<0.001
Number of chronic diseases				0.091
0	–	–	1.00 (Ref)	–
1	−0.039	0.07	0.962 (0.838–1.104)	0.577
≥2	0.234	0.121	1.264 (0.997–1.603)	0.053
Poor self-rated health				
No	–	–	1.00 (Ref)	–
Yes	0.517	0.179	1.676 (1.181–2.380)	0.004
Depressive symptoms (CES-D ≥16)				
No	–	–	1.00 (Ref)	–
Yes	0.678	0.151	1.969 (1.465–2.646)	<0.001
Anxiety symptoms (SAS ≥50)				
No	–	–	1.00 (Ref)	–
Yes	0.077	0.158	1.080 (0.792–1.473)	0.626
Sleep disturbance (PSQI >7)				
No	–	–	1.00 (Ref)	–
Yes	0.184	0.079	1.202 (1.030–1.404)	0.02

This table presents the results of the multivariable logistic regression analysis for factors associated with AD8 positivity. Due to missing data in certain covariates (marital status, smoking status, drinking status, and number of chronic diseases), the multivariable logistic regression model was performed on a complete-case basis, comprising 7,293 participants. Adjusted odds ratios (aORs), 95% confidence intervals (CIs), regression coefficients (*β*), and standard errors (SEs) are reported.

aOR, Adjusted Odds Ratio; CI, Confidence Interval; SE, Standard Error; β, Regression Coefficient; Ref, Reference category.

### 3.2 Univariate logistic regression results

Univariate logistic regression analyses were performed among the 7,601 valid participants to screen for potential predictors (Table 2). Thirteen of the fourteen candidate variables—spanning demographic, behavioral, and psychological domains—were significantly associated with AD8 positivity in the univariate analyses (*p* < 0.05); only household economic status did not show statistical significance. These univariate results informed the subsequent multivariable analysis.

### 3.3 Multivariable logistic regression results

All fourteen candidate variables, including the thirteen significant in univariate analysis and household economic status, were simultaneously incorporated into the multivariable logistic regression model on a complete-case basis (*N* = 7,293). Nine variables were independently associated with AD8 positivity (Table 3; Figure 2).

**Figure 2:**
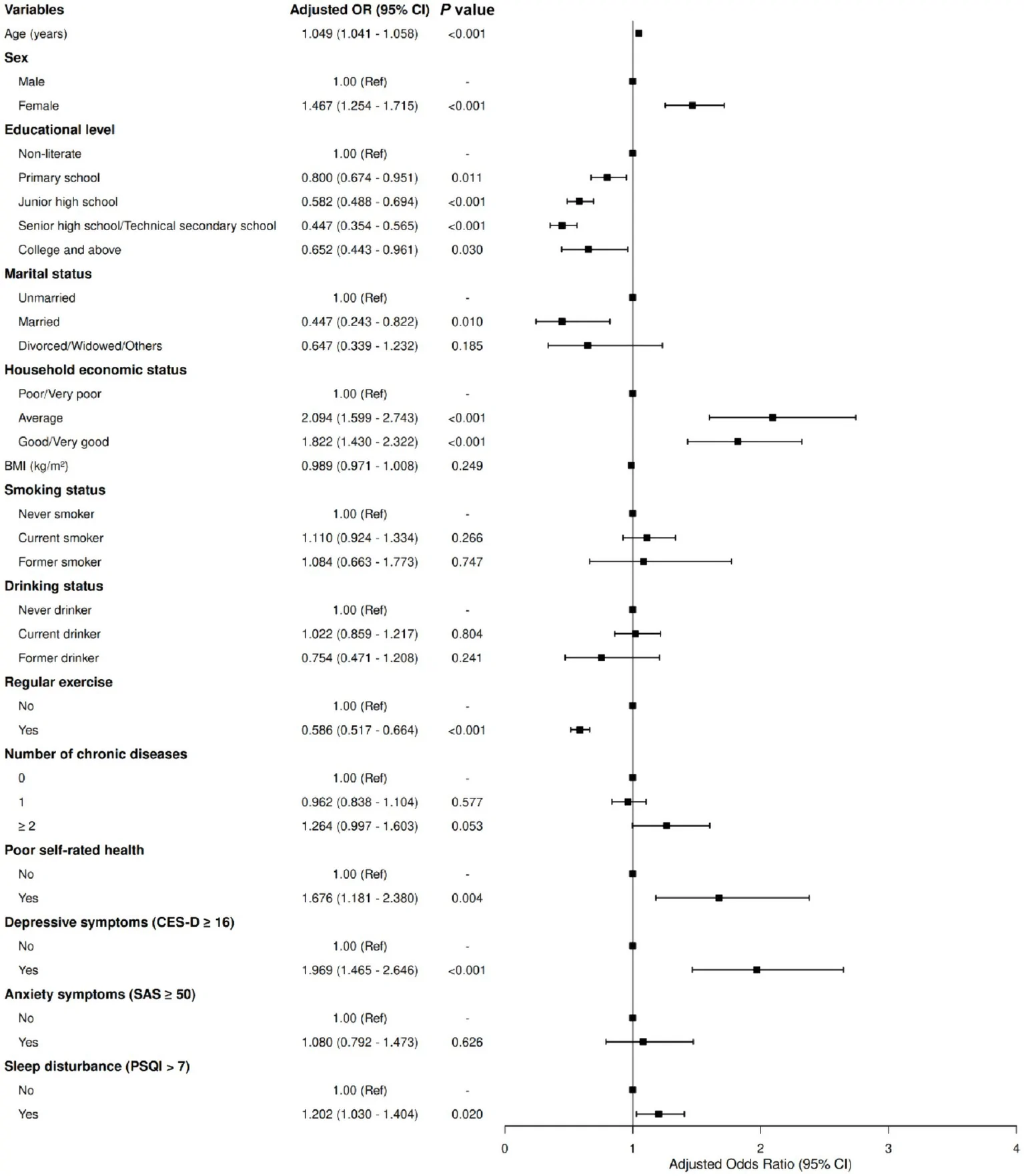
Forest plot of independent factors associated with AD8 positivity in community-dwelling middle-aged and older adults. Forest plot showing adjusted odds ratios and 95% confidence intervals for factors independently associated with AD8 positivity.

Among the identified health and psychological risk factors, depressive symptoms and poor self-rated health demonstrated the most prominent positive associations with AD8 positivity (adjusted odds ratio [aOR] = 1.97 and 1.68, respectively, both *p* < 0.05). Advanced age, female sex, and sleep disturbances were also identified. Additionally, being married was negatively associated with AD8 positivity compared to being unmarried. Conversely, regular physical activity and higher educational attainment served as significant protective factors. Notably, a dose–response pattern was observed regarding educational attainment, where higher educational attainment, particularly from primary school through senior high school, was associated with progressively lower odds of AD8 positivity compared with the non-literate group, although the magnitude of this inverse association attenuated slightly for those with a college education and above.

An unexpected finding was observed regarding household economic status. After controlling for confounders such as educational attainment and health status in the multivariable model, household economic status transitioned into a highly significant independent predictor, despite lacking significance in the univariate analysis. Participants with average (aOR = 2.09) or good/very good (aOR = 1.82) economic status unexpectedly exhibited higher odds of AD8 positivity compared with the poor/very poor reference group. Furthermore, variance inflation factor (VIF) diagnostics were performed for all variables in the model. The detailed VIF values and the evaluation of potential multicollinearity—including the specific assessment of an elevated VIF for marital status—are presented in Supplementary Table 1.

## 4 Discussion

Based on the baseline data of the Taierzhuang cohort, this cross-sectional study investigated factors associated with AD8 positivity among community-dwelling middle-aged and older adults. Our analysis revealed that multiple demographic, lifestyle, and health-related factors were independently associated with AD8 positivity among this population. These findings provide meaningful evidence for the early identification and targeted management of individuals at elevated cognitive risk in community settings.

### 4.1 Main findings

With the accelerating population aging in China, cognitive impairment has become an increasingly important public health challenge, imposing substantial burdens on individuals, families and healthcare systems (28, 29). Early identification of individuals at increased cognitive risk is therefore an important component of community-based healthy aging strategies. However, large-scale cognitive assessment in primary healthcare settings remains challenging because commonly used instruments such as the MMSE and MoCA require trained personnel and relatively long administration times (30).

In the present study, the AD8 questionnaire was used as a rapid screening tool to identify individuals with AD8 positivity in the community. Based on a large sample of community-dwelling middle-aged and older adults, we comprehensively evaluated the associations of demographic characteristics, lifestyle behaviors and health-related factors with AD8 positivity. The results demonstrated that a combination of multidimensional factors independently contributed to AD8 positivity. These findings are generally consistent with existing evidence regarding factors related to cognitive decline and highlight the multidimensional nature of cognitive health in community populations (1). The identified factors are readily obtainable through routine community surveys and health assessments, suggesting that they may be useful for identifying potentially vulnerable populations and informing targeted health management strategies in primary care and public health practice.

### 4.2 Demographic factors

In the current study, advanced age, female sex, and lower educational attainment were significantly associated with an increased risk of AD8 positivity (31, 32). The association between advanced age and cognitive decline may be driven by underlying mechanisms such as age-related neurodegenerative changes, cumulative cerebrovascular damage, and a progressive decline in cognitive reserve over time (33). From a neurobiological perspective, the progressive depletion of cognitive reserve over time impairs neural compensation and synaptic plasticity, making older adults more vulnerable to expressing subjective cognitive functional decline when confronted with age-related neurodegenerative changes. Regarding educational attainment, the cognitive reserve theory suggests that higher education helps build robust neural networks and cognitive paradigms, which may buffer against early neuropathological damage and delay the clinical manifestation of cognitive impairment.

### 4.3 Lifestyle factors

Regular physical activity was identified as an independent protective factor against AD8-defined high cognitive risk (34). Mechanistically, physical exercise may preserve cognitive function by upregulating neurotrophic factors, promoting neurogenesis, and improving cerebral blood flow, thereby enhancing synaptic plasticity and reducing the rate of neurodegeneration (35).

In the univariate analysis (Table 2), some lifestyle and physical factors, specifically BMI, smoking status, and drinking status, showed counterintuitive negative associations with AD8 positivity. However, it is essential to distinguish between these crude associations and the adjusted results. These unadjusted associations did not persist in the fully adjusted multivariable model (Table 3), suggesting they may partly reflect demographic confounding rather than true protective effects. For instance, in our cohort, current smokers and drinkers were predominantly males, who generally exhibited a lower rate of AD8 positivity compared to older, non-literate females. Once adjusted for age, sex, and other covariates, these associations were no longer significant, suggesting they may partly reflect confounding. Furthermore, the lack of significant association for BMI might be influenced by reverse causality—where early-stage cognitive decline or related morbidities affect body weight—as well as the inherent limitations of utilizing a single baseline measurement of exposure.

### 4.4 Psychological and health factors

Psychological and health-related indicators, including depressive symptoms, sleep disturbances, and poor self-rated health, were positively associated with AD8 positivity (3, 4). The link between depression and cognitive risk could be explained by abnormalities in the hypothalamic–pituitary–adrenal (HPA) axis and chronic neuroinflammation, which may negatively impact hippocampal volume and function (36). One possible explanation for the impact of sleep is that sleep disturbances may impair the clearance of amyloid-beta proteins via the glymphatic system and disrupt memory consolidation processes during the sleep cycle (37). Poor self-rated health further reflects the cumulative burden of these adverse health conditions on cognitive function (38).

### 4.5 Economic status

An unexpected finding of the present study was that participants reporting average or good household economic status exhibited a higher likelihood of AD8 positivity compared with those reporting poor economic status. This observation appears inconsistent with the conventional epidemiological evidence suggesting that socioeconomic advantage is generally associated with better cognitive health and a lower risk of dementia (39). Therefore, this finding should be interpreted cautiously.

Several explanations may account for this result. One primary explanation is that AD8 is a subjective screening instrument that relies largely on self-perceived cognitive changes rather than objective neuropsychological assessment. Individuals with higher socioeconomic status often possess greater health literacy, stronger awareness of cognitive health, and more frequent health-seeking behavior. Aligning with the cognitive awareness hypothesis, they may therefore be more susceptible to subtle cognitive changes and more likely to report functional decline or memory problems, which raises the likelihood that they may test positive on the AD8 (40). In contrast, individuals with lower socioeconomic status may underrecognize or underreport cognitive symptoms, leading to potential differential reporting bias.

Another contributing factor is that household economic status in the present study was assessed using self-reported subjective categories rather than objective indicators such as income, wealth, or occupational status. Previous studies have shown that subjective socioeconomic measures may not fully correspond to actual material resources and can be influenced by individual expectations, social comparison, and psychological factors (41). Therefore, the observed association may partly reflect differences in perception and reporting behavior rather than true socioeconomic gradients in cognitive function.

Furthermore, because this study employed a cross-sectional design, reverse causality and residual confounding cannot be excluded. Even after controlling for a number of demographic, lifestyle, and health-related factors, the observed correlation may still be impacted by unmeasured traits such as social participation, healthcare utilization, family support, cognitive reserve, and health-seeking behavior (5, 42). Moreover, the relatively high AD8-positive rate in this community population may amplify the impact of subjective reporting characteristics on the observed associations.

When considered collectively, the unexpected positive correlation between AD8 positivity and self-reported greater household economic status must be interpreted carefully. This result may be due to residual confounding in cross-sectional self-reported socioeconomic measures, increased health literacy awareness, and differential reporting bias rather than a true biological gradient. Furthermore, to elucidate the substantial change in the association between economic status and AD8 positivity between unadjusted and adjusted models, we conducted sequentially adjusted models (Supplementary Table 2). The results demonstrated that the association, which was masked in the unadjusted model, was highly sensitive to covariate adjustment. The independent effect of economic status only became highly significant after sequentially adjusting for confounding factors across the models. This substantial change suggests that the unadjusted association may have been obscured by complex confounding from demographic factors (such as educational level), as well as the inherent non-collapsibility of odds ratios in logistic regression, emphasizing the multifaceted nature of socioeconomic influences. To elucidate the nature of this connection, more longitudinal studies that include comprehensive socioeconomic variables and objective cognitive evaluations are necessary. As a result, no causal inference should be made and this finding should be regarded as hypothesis-generating.

### 4.6 Possible causes of the high positive rate of AD8

The detection rate of AD8 positivity was relatively high in the present study. It should be noted that the AD8 is a highly sensitive screening instrument designed to identify individuals with potential cognitive abnormalities at an early stage, rather than to establish a clinical diagnosis of cognitive impairment or dementia. Previous community-based screening studies using the AD8 with a cut-off of ≥2 in Chinese populations have reported varying positivity rates, frequently ranging from 40% to over 60% depending on the specific demographic composition, which are inherently distinct from the prevalence of clinically diagnosed dementia (15, 16). Furthermore, while previous community-based studies frequently report elevated positivity rates with informant-administered AD8, self-administered forms may capture a broader range of subjective health concerns, potentially contributing to higher positive screening rates. Therefore, our strikingly high prevalence (>80%) must be interpreted within the context of our specific administration method (self-report) and highly vulnerable demographic profile (predominantly older and non-literate populations). Therefore, the proportion of individuals classified as AD8 positive is generally expected to be substantially higher than the prevalence of clinically diagnosed cognitive disorders based on comprehensive neuropsychological assessments or formal diagnostic criteria. Consequently, direct comparisons with clinically diagnosed dementia prevalence should be interpreted cautiously.

In addition, the participants in this study were predominantly community-dwelling middle-aged and older adults with a relatively advanced age structure. Age is one of the most consistently reported factors associated with cognitive decline (43). Furthermore, the study population generally had relatively low educational attainment, and a considerable proportion of participants experienced depressive symptoms, sleep disturbances, and chronic health conditions, all of which have been reported to be associated with poorer cognitive performance (3, 4). These characteristics may have collectively contributed to the high proportion of AD8 positivity observed in this population.

Another possible explanation is related to the characteristics of the AD8 itself. The AD8 includes several items assessing subjective changes in memory, judgment, and daily functioning. In community-based surveys, participants may be more likely to perceive and report subtle cognitive changes that have not yet reached the threshold of clinically diagnosed cognitive impairment (44). This screening pattern is consistent with existing community-based epidemiological studies among middle-aged and older Chinese adults, where brief subjective screening tools frequently yield relatively high positivity rates due to self-reported functional complaints, psychological distress, and the age-related accumulation of somatic burdens (15, 16, 40). Consequently, subjective concerns regarding cognitive function may further increase the positive screening rate. Importantly, nearly 50% of our participants were non-literate. Subjective cognitive screening tools like the AD8 may be less suitable for underserved and low-literacy populations due to varying health literacy and cultural interpretations of questionnaire items. It is crucial to distinguish between a positive screening result and a clinical diagnosis of cognitive impairment. Because our study lacks validation from objective neuropsychological assessments, we cannot definitively prove an inflated false-positive rate or determine the exact extent of potential measurement bias. Nonetheless, as has been observed with other standard cognitive assessment tools in underserved populations, this limitation highlights the need for caution when interpreting self-reported cognitive screening results. Therefore, AD8 positivity should be interpreted as an indicator of elevated cognitive risk rather than a diagnosis of cognitive impairment or dementia.

Although the proportion of AD8 positive individuals was relatively high, the primary objective of this study was not to estimate the prevalence of cognitive impairment, but to explore factors associated with AD8 positivity in a community population (15). From a public health perspective, identifying characteristics associated with AD8 positivity may help primary healthcare providers recognize potentially vulnerable groups and facilitate targeted cognitive health promotion and early screening strategies in community settings.

### 4.7 Strengths and limitations

The present study has several strengths. A major strength is that our research was conducted in a large community-based population, providing sufficient statistical power to examine factors associated with AD8 positivity. Additionally, a wide range of demographic characteristics, lifestyle factors, psychological indicators, and health-related variables were simultaneously considered, allowing a comprehensive assessment of potential correlates of AD8 positivity in community settings. Moreover, all variables included in the analysis were readily obtainable through routine questionnaires and community health surveys, enhancing the practical relevance of the findings for primary healthcare and public health management. Furthermore, the study specifically focused on AD8-defined cognitive risk in community-dwelling populations, which may contribute to the early identification of vulnerable groups and support the development of targeted prevention strategies.

Several limitations should also be acknowledged. The primary limitation is that, owing to the cross-sectional design, temporal relationships between associated factors and AD8 positivity could not be determined; therefore, causal inferences cannot be established (45). To confirm the directionality of these correlations, more prospective cohort studies are required. Moreover, several variables, including lifestyle behaviors, self-rated health, depressive symptoms, and sleep status, were collected through self-reported questionnaires, which may be subject to recall bias and reporting bias. In addition, although we adjusted for a comprehensive set of predefined variables, residual confounding from important unmeasured factors—such as hearing impairment, cardiovascular disease history, specific medication use, and levels of social participation—cannot be completely ruled out, as these indicators were not captured in the baseline survey. Another constraint to consider is that AD8 is a screening instrument rather than a clinical diagnostic tool for cognitive impairment or dementia. Therefore, the outcome of this study reflects AD8 positivity rather than clinically confirmed cognitive disorders. The relatively high positive rate observed in the present study should be interpreted within the context of the high sensitivity of the AD8 screening tool. Lastly, the results may not be as applicable to populations in other geographic areas or sociocultural contexts because the participants were drawn from a single community-based cohort in one area of China. To corroborate these findings, more research with populations from various geographic locations and social backgrounds is necessary.

## 5 Conclusion

In this cross-sectional study of community-dwelling middle-aged and older adults, age, female sex, marital status, lower educational attainment, lack of regular physical activity, poor self-rated health, depressive symptoms, sleep disturbances, and household economic status were independently associated with AD8 positivity. Rather than merely reflecting a static profile of individual characteristics, these findings highlight the critical value of the AD8 as a sensitive community screening instrument for the early detection of cognitive vulnerability. These findings may provide evidence for community cognitive health surveillance and targeted risk management strategies among middle-aged and older adults.

## Data Availability

The raw data supporting the conclusions of this article will be made available by the authors, without undue reservation.
