# Supplementary material for: Factors associated with AD8-defined high cognitive risk in community-dwelling middle-aged and older adults: a cross-sectional study
Source: Front Public Health. 2026 Oct 29;14:1927564. doi: 10.3389/fpubh.2026.1927564 (PMC13754541; doi:10.3389/fpubh.2026.1927564)
Supplement: Supplementary file 1 [file Table_1.DOCX]

**Supplementary Table 1. Variance inflation factor (VIF) diagnostics for variables included in the multivariable logistic regression model (*N* = 7,293).**

| **Variables** | **Tolerance** | **VIF** |
| --- | --- | --- |
| **Age (years)** | 0.64 | 1.562 |
| **Sex** | 0.602 | 1.661 |
| **Educational level** | 0.648 | 1.544 |
| **Marital status** | 0.102 | 9.82 |
| **Household economic status** | 0.297 | 3.37 |
| **BMI (kg/m²)** | 0.905 | 1.105 |
| **Smoking status** | 0.731 | 1.368 |
| **Drinking status** | 0.691 | 1.446 |
| **Regular exercise** | 0.916 | 1.092 |
| **Number of chronic diseases** | 0.906 | 1.104 |
| **Poor self-rated health** | 0.926 | 1.08 |
| **Depressive symptoms (CES-D ≥ 16)** | 0.812 | 1.231 |
| **Anxiety symptoms (SAS ≥ 50)** | 0.852 | 1.173 |
| **Sleep disturbance (PSQI > 7)** | 0.889 | 1.125 |

Note: VIF diagnostics were conducted for the predictors included in the multivariable logistic regression model. The elevated VIF observed for marital status (VIF = 9.82) was thoroughly investigated. This inflation is a structural artifact arising directly from the dummy coding of its categories relative to the small proportion of the unmarried reference group, rather than true substantive multicollinearity with other distinct variables. Further diagnostics confirmed that the standard errors for the marital status categories remained constrained, and the model's key estimates and standard errors for all other covariates remained completely stable. Therefore, this structural artifact does not compromise the validity or stability of the multivariable model.

**Supplementary Table 2. Sequentially adjusted models for the association between household economic status and AD8 positivity (N=7,293).**

| **Variables** | **Model 1** | **Model 2** | **Model 3** |
| --- | --- | --- | --- |
| **Household economic status** |  |  |  |
| Poor/Very poor | 1.00 (Ref) | 1.00 (Ref) | 1.00 (Ref) |
| Average | 0.966 (0.758 - 1.230), P = 0.778 | 1.892 (1.455 - 2.462), P < 0.001 | 2.094 (1.599 - 2.743), P < 0.001 |
| Good/Very good | 1.101 (0.883 - 1.373), P = 0.394 | 1.589 (1.255 - 2.012), P < 0.001 | 1.822 (1.430 - 2.322), P < 0.001 |

Note: Model 1: Unadjusted model (only includes household economic status). Model 2: Adjusted for demographic characteristics (age, sex, educational level, and marital status). Model 3: Fully adjusted model (adjusted for variables in Model 2 plus BMI, smoking status, drinking status, regular exercise, number of chronic diseases, self-rated health, depressive symptoms, anxiety symptoms, and sleep disturbances).
